# Online Detection of Multiple Stimulus Changes Based on Single Neuron Interspike Intervals

**DOI:** 10.3389/fncom.2019.00069

**Published:** 2019-10-01

**Authors:** Lena Koepcke, K. Jannis Hildebrandt, Jutta Kretzberg

**Affiliations:** ^1^Computational Neuroscience, Department of Neuroscience, University of Oldenburg, Oldenburg, Germany; ^2^Cluster of Excellence “Hearing4All”, University of Oldenburg, Oldenburg, Germany; ^3^Auditory Neuroscience, Department of Neuroscience, University of Oldenburg, Oldenburg, Germany

**Keywords:** change point analysis, spike train analysis, spike coding, burst detection, interspike interval, ROC, cricket, AN2

## Abstract

Nervous systems need to detect stimulus changes based on their neuronal responses without using any additional information on the number, times, and types of stimulus changes. Here, two relatively simple, biologically realistic change point detection methods are compared with two common analysis methods. The four methods are applied to intra- and extracellularly recorded responses of a single cricket interneuron (AN2) to acoustic simulation. Solely based on these recorded responses, the methods should detect an unknown number of different types of sound intensity in- and decreases shortly after their occurrences. For this task, the methods rely on calculating an adjusting interspike interval (ISI). Both simple methods try to separate responses to intensity in- or decreases from activity during constant stimulation. The Pure-ISI method performs this task based on the distribution of the ISI, while the ISI-Ratio method uses the ratio of actual and previous ISI. These methods are compared to the frequently used Moving-Average method, which calculates mean and standard deviation of the instantaneous spike rate in a moving interval. Additionally, a classification method provides the upper limit of the change point detection performance that can be expected for the cricket interneuron responses. The classification learns the statistical properties of the actual and previous ISI during stimulus changes and constant stimulation from a training data set. The main results are: (1) The Moving-Average method requires a stable activity in a long interval to estimate the previous activity, which was not always given in our data set. (2) The Pure-ISI method can reliably detect stimulus intensity increases when the neuron bursts, but it fails to identify intensity decreases. (3) The ISI-Ratio method detects stimulus in- and decreases well, if the spike train is not too noisy. (4) The classification method shows good performance for the detection of stimulus in- and decreases. But due to the statistical learning, this method tends to confuse responses to constant stimulation with responses triggered by a stimulus change. Our results suggest that stimulus change detection does not require computationally costly mechanisms. Simple nervous systems like the cricket's could effectively apply ISI-Ratios to solve this fundamental task.

## 1. Introduction

Has anything relevant changed in the sensory environment? This question is so essential for all organisms that the major task of sensory systems is to reflect behaviorally relevant stimulus changes in their neuronal activity. While vertebrates usually rely on large populations of neurons in each processing step, invertebrates have to accomplish stimulus detection with few individual neurons. This study is focused on the detection of multiple stimulus changes based on the spike train of a single invertebrate neuron. Four different change point (CP) detection methods are compared, which rely solely on information available to the nervous system—one or two interspike intervals (ISI) preceding a spike.

Detecting relevant changes in the neuronal activity (Ellaway, 1978; Legéndy and Salcman, 1985; Goense and Ratnam, 2003; Gourévitch and Eggermont, 2007; Levakova et al., 2015) was frequently used for the detection of stimulus changes (Ellaway, 1978; Goense and Ratnam, 2003; Levakova et al., 2015; Koepcke et al., 2016), e.g., based on the CUSUM (CUmulative SUM) method (Ellaway, 1978; Koepcke et al., 2016). A common application is the identification of the response latency to stimulus onset/offset of a single stimulus (Oram and Perrett, 1992; Ratnam et al., 2003; Levakova et al., 2015). This requires a good estimation of the starting point of the neural response. To achieve a better estimation, many CP detection methods use the entire recording and/or averaged data over responses to multiple stimulus repetitions. Approaches that rely on spike patterns occurring after the stimulus change that would not be readily available to the nervous system, are called “offline.” In contrast, “online” algorithms identify a CP in the stimulus based on data that is updated in every time step of the experimental recording. Since offline algorithms use a larger amount of data for making a decision, they are usually more accurate and detect changes with shorter delay than online methods (Aminikhanghahi and Cook, 2017).

Another approach of CP detection methods is the detection of “bursts” and/or “pauses” in spike trains (Legéndy and Salcman, 1985; Cocatre-Zilgien and Delcomyn, 1992; Gourévitch and Eggermont, 2007; Tokdar et al., 2010; Kapucu et al., 2012). The goal of this approach is to analyze the relevance of bursts and response gaps in sensory coding. Most published methods for the detection of bursts or pauses in spike trains utilize offline-algorithms. Especially the identification of bursts has received a lot of attention in the neuroscientific literature (Legéndy and Salcman, 1985; Cocatre-Zilgien and Delcomyn, 1992; Xu et al., 1999; Pauluis and Baker, 2000; Gourévitch and Eggermont, 2007; Pasquale et al., 2010; Tokdar et al., 2010; Kapucu et al., 2012; Ko et al., 2012). The simplest approach for detecting a burst onset is to apply a simple threshold to the interspike interval or spike frequency (Kepecs and Lisman, 2004; Marsat and Pollack, 2006), sometimes followed by hypothesis testing (Cocatre-Zilgien and Delcomyn, 1992). These thresholds are usually determined by the analysis of the (transformed) interspike interval (ISI) histogram (Legéndy and Salcman, 1985; Gourévitch and Eggermont, 2007; Ko et al., 2012). ISI distributions also provide the basis for the published methods for the detection and analysis of intervals with a particularly low firing rate (“pauses”; Elias et al., 2007; Yartsev et al., 2009; Ko et al., 2012; Gärtner et al., 2017).

In this study, we propose two biologically realistic CP detection methods that rely on interspike intervals, which we compare to two less plausible but frequently used approaches. We apply these four methods to the responses of single first order auditory interneurons (AN2) in crickets. Crickets use their auditory system to find mating partners (“low frequency,” 3–8 kHz, Wyttenbach et al., 1996) and to avoid predators (bat echolocation, 15–80 kHz, Hoy, 1992). For both auditory tasks, crickets have to recognize intensity changes, which can be either in- or decreases in the sound intensity.

The crickets' auditory system can perform complex analysis of the acoustic environment with a relatively simple structure. Sounds are received via the tympana in the forelegs, where ~70 auditory receptor neurons are located (Young and Ball, 1974). The first stage of auditory processing comprises only two bilateral pairs of ascending interneurons (AN1 and AN2) that are directly connected to the brain. AN2 is mainly specialized in the high frequencies (ultrasound) of the echolocation of hunting bats. Bursts in AN2 trigger an avoidance behavior of the cricket (Marsat and Pollack, 2006). However, AN2 also receives excitation and inhibition at the dominant frequency (3–8 kHz) of the calling song (Moiseff and Hoy, 1983).

In AN2, neuronal activity is usually increased, when the sound intensity rises and decreased during intensity declines (Hildebrandt et al., 2011). However, external stimuli are not the only cause for changes in the neuronal activity. They can also result from internal sources including adaptation or intrinsic noise. Therefore, the system has to find a balance between missing perceptions and oversensitive interpretation of the neuronal activity. Because a change of an acoustic signal is reflected in the activity of a single neuron (AN2), the auditory system has to rely on individual spike times and interspike intervals (ISI). In this study, we analyzed recordings of AN2 interneurons stimulated with varying sound intensities. The neurons were stimulated with their preferred frequency (16 kHz, echolocation) or, for comparison, the fundamental frequency of the calling song (3 kHz).

The CP detection methods compared in this study had to face the following specific challenges: (1) The activity of only a single neuron was available. (2) Only online-methods were considered, which are able to make their decisions based on data available to the neuronal system. (Neither spikes occurring after each time point nor averaged data was used.) (3) The methods had to detect an unknown number of stimulus changes. (4) Different types of stimulus changes (i.e., intensity in- and decreases) had to be detected.

We selected four CP detection methods, which are based on the analysis of ISIs: (1) The Moving-Average method, also known as the Rate-Change Method (Baker and Gerstein, 2001; Levakova et al., 2015; Koepcke et al., 2016), is a classical approach for detecting changes in a time series. It compares the inverse of the actual ISI with the mean and the standard deviation of the neuronal activity in a previous reference interval. This procedure is a standard method to test if a value belongs to the same distribution as some reference values. (2) The Pure-ISI method, the simplest biologically plausible CP detection approach we could think of, compares the actual ISI with a given threshold, without comparing it to previous activity. (3) The ISI-Ratio method we propose assumes that stimulus changes result in multiplicative changes of the neuronal activity. It applies a threshold to the ratio of actual and previous ISI. (4) The classification method detects stimulus changes by identifying combinations of actual and previous ISI. The underlying assumption of this powerful, but computationally expensive method is that nervous systems can adapt their responses to stimulus statistics.

Hence, two of these methods, Pure-ISI and classification, rely on absolute ISI values, while the other two methods use relative changes from previous activity. All four of our methods critically depend on a predefined threshold as a criterion for CP detection. The tradeoff is that increasing the threshold increases the probability of missing stimulus changes, whereas it decreases the probability for false positives, which are not caused by a stimulus change. We apply the receiver operating characteristic (ROC) to analyze different thresholds for the evaluation of the CP detection methods.

## 2. Methods

All applied methods, data analysis and figures were programmed and created with MATLAB (R2017a, The MathWorks, Inc.).

### 2.1. Experiments

We adopted neuronal data published by Hildebrandt et al. (2011), which was not optimized for the development of our methods, but rather was used as an example for available data. In the present study, data from 21 crickets (13 females and 8 males) (*Teleogryllus leo*) were analyzed, of which 9 animals were used for intracellular recordings and 12 animals for extracellular recordings. For technical details of the experiments, refer to Hildebrandt et al. (2011).

**Figure 1:**
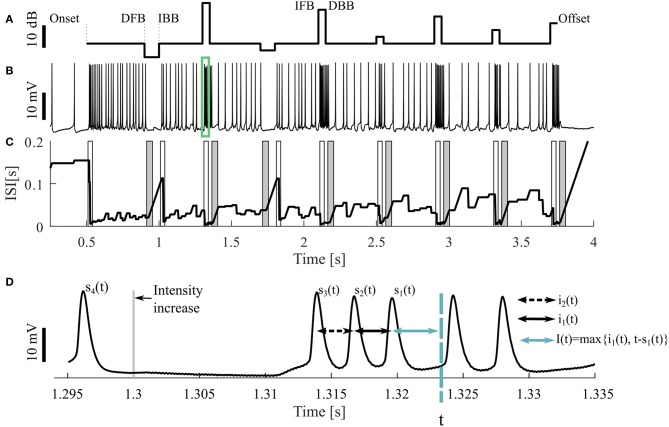
Sample stimulus and neuronal response. **(A)** Stimulus pattern consisting of DFB (decrease from baseline), IBB (increase back to baseline), IFB (increase from baseline), and DBB (decrease back to baseline), as well as **(B)** one resulting intracellular recording. The cell was stimulated with a 16 kHz tone with different intensities, the base intensity was 98 SPL. The acoustic stimulation started at the time of 0.5 s and ended at 3.75 s of the 4.25 s long recording. The green rectangle indicates the period shown in **(D)**. **(C)** Adjusting ISI (*I*_*a*_(*t*), Equation 3), The light (dark) gray areas represent the periods when a response to an intensity increase (decrease) was expected. If a CP was detected in this period, it was classified as true positive. **(D)** A blow-up of the raw voltage data in the range marked by the green rectangle in **(B)**. For the time point *t* the three previous spike times [*s*_1_(*t*), *s*_2_(*t*), *s*_3_(*t*)] as well the two previous ISIs [*i*_1_(*t*), *i*_2_(*t*)] and an example of the adjusting ISI are illustrated (section 2.2.2).

The carrier frequency (pitch) of 16 or 3 kHz of a stimulus pattern remained constant during each experiment (Figure 1A). The ultrasound (16 kHz) represented the frequency of the bat echolocation, whereas the low frequency sound (3 kHz) corresponded to the fundamental frequency of the calling song *Teleogryllus leo* (Rothbart and Hennig, 2012). Each stimulus pattern had a base sound intensity and consisted of rectangular positive and negative steps relative to the baseline intensity (Figure 1A; 1.3 and 0.9 s). The positive steps lasted 50 ms and were 2–18 dB louder than the baseline intensity. The duration of the negative steps was 100 ms and the intensity was 2–5 dB lower. Intensity steps were presented every 400 ms, leading to 300 or 350 ms of baseline intensity between the steps.

The neural activity was recorded 500 ms before and after stimulation (Figure 1A). Consequently, three different kinds of intensity increases were applied: sound onset (onset; at 0.5 s), increases from baseline (IFB; e.g., at 1.3 s) and increases back to baseline (IBB; e.g., at 1 s). Correspondingly, intensity decreases consisted of: sound offsets (offset; at 3.75 s), decreases from baseline (DFB; e.g., at 0.9 s), and decreases back to baseline (DBB; e.g., at 1.35 s). However, since positive intensity steps occurred more frequently than negative steps, onsets and offsets, the most common stimulus changes were IFBs and DBBs. The baseline sound level varied between 75 and 98 SPL. Each recording consisted of 10 trials, identical repetitions of the same stimulus protocol, interrupted by breaks of 1,500 ms. All 10 trials were used for analysis. Between one and nine different stimulus protocols were used for each animal. In total, we analyzed 9 intracellular recordings with 16 kHz stimulation as our standard data set. Additionally, 11 intracellular recordings with 3 kHz and 73 extracellular recordings (16 kHz: 43, 3 kHz: 30, in 12 animals) were analyzed to test the robustness of our methods.

### 2.2. Prerequisites for Stimulus Change Point Detection Methods

The goal of the four change point (CP) detection methods compared in this study is to detect the responses to all stimulus intensity changes (increases as well as decreases, Figure 1A) based on the recorded neuronal data. Since an intensity increase typically resulted in an increase of the neural activity (Hildebrandt et al., 2011), while an intensity decrease reduced the neuronal activity, the intensity in- and decreases were analyzed independently. The time points of stimulus changes are called “(stimulus) change points” (CPs), the outcomes of the CP detection methods are called “putative CPs” and the time points, which fulfill the criteria of a putative CP before the application of restriction criteria, are called “threshold crossings.” The restriction criteria for a putative CP are described below.

#### 2.2.1. Putative Change Points and True and False Positives

The classification of a putative CP as a correctly or falsely detected CP (true and false positive) relies on the time period, during which a neuronal response to a stimulus CP can be expected. To determine this period, the distribution of the response latencies was estimated (Supplementary Material).

The time interval after a stimulus change, during which a putative CP is classified as true positive is called accepted time range (*T*_accept_). The accepted time range for intensity increases was set to [10, 40] ms and for intensity decreases to [15, 55] ms (Supplementary Material).

Because the analyzed data were continuous time series, multiple putative CPs could be detected in this time range. If this was the case, only the first putative CP was classified as true positive (TP) and the others as false positives (FP).

**Figure 2:**
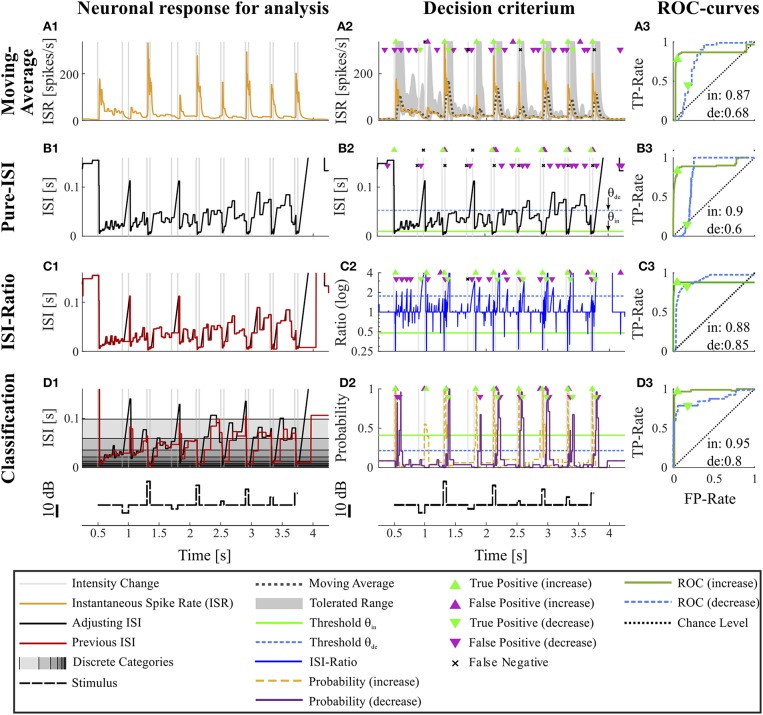
Overview of all methods. The example data is identical to the data in Figure 1. The first column **(A1–D1)** shows the main idea of the first step of each method. The second column **(A2–D2)** depicts the basis of decision when a CP was found and indicates the resulting CPs and missed changes. Here, the thresholds were chosen to yield a false positive rate of 0.05 (increases) and 0.15 (decreases). The third column **(A3–D3)** shows the ROC-curves for this particular experiment and specific methods' parameter settings (section 2.4) for the detection of intensity increases and decreases. The triangles indicate the performance for the thresholds applied in the second column. The black line represents the chance level. The rows of the figure represent the CP detection methods: **(A1–A3)** Moving Average method: **(A1)** Instantaneous spike rate (section 2.3.1, Equation 5); **(A2)** Instantaneous spike rate together with moving average and the corresponding multiple of standard deviation (Equation 6). Here, a moving average window size of 100 ms was applied and the multiple of standard deviation was 13.7 for intensity increases and 1.65 for decreases. **(B1–B3)** Pure ISI method: **(B1,B2)** Sketch of the method (section 2.3.2). The adjusting ISI (Equation 3) is illustrated together with a specific pair of thresholds (*θ*_*in*_ = 30 ms, *θ*_*de*_ = 77 ms Equation 10). **(C1–C3)** ISI-Ratio method: **(C1)** Adjusting and previous ISI with weight 0 (*I*_*a*_(*t*), *I*_*pre*_(*t*, 0)) (section 2.2.2 Equations 3, 4); **C2** Ratio of adjusting and previous ISI with a logarithmically scaled y-axis. The thresholds in this example were *θ*_*in*_ = 0.7 and *θ*_*de*_ = 1.4. **(D1–D3)** Classification method: **(D1)** Adjusting and previous ISI. The different background gray-levels indicate ten different categories (section 2.3.4, Equation 14); **(D2)** Probabilities of intensity increase and decrease with thresholds *θ*_*in*_ = 0.17 and *θ*_*de*_ = 0.11.

If the threshold for CP detection stayed crossed for several consecutive time points, only the first threshold crossing was considered as a putative CP. Therefore, it could happen that CPs were missed when the corresponding threshold crossings occurred within a longer period (e.g., more than 40 ms) of continuously crossed threshold. This caused a theoretical problem when applying the ROC curve as an evaluation technique. It was necessary that lower sensitivity resulted in high numbers of TPs and FPs. If threshold crossings occurred successively longer than the accepted time range (|*T*_accept_|), the algorithm was reset, allowing detection of further putative CPs. However, it was not allowed to detect several putative CPs between two spikes. With this modification the number of FPs increased with the number of TPs (e.g., Figures 2A–D3) when relaxing the threshold criterion for CP detection.

#### 2.2.2. Interspike Interval (ISI)

All four methods compared in this study rely on the analysis of adjusting ISIs. For the spike times *t*_1_, *t*_2_, …, *t*_*n*_ in one trial (response to one representation of a stimulus protocol), with *s*_1_(*t*) the previous spike time of any time point *t* > *t*_2_, *s*_2_(*t*) the second last and *s*_*i*_(*t*) the *i*-th last spike time (Figure 1D) are defined by

(1) $$\begin{array}{l} s_{1}\left(t\right)=\max\left\{t_{j}|t_{j}\leq t\right\},\;s_{2}\left(t\right)=\max\left\{t_{j}|t_{j}<s_{1}\left(t\right)\right\},\\ s_{i}\left(t\right)=\max\left\{t_{j}|t_{j}<s_{i-1}\left(t\right)\right\}. \end{array}$$

The actual ISI at time point *t* (*i*_1_(*t*)) is the time difference between the two previous spike times. Correspondingly, the previous ISI (*i*_2_(*t*)) is the difference between the second and third last spike time:

(2) $$\begin{array}{l} i_{1}\left(t\right)=s_{1}\left(t\right)-s_{2}\left(t\right),\;i_{2}\left(t\right)=s_{2}\left(t\right)-s_{3}\left(t\right),\\ i_{3}\left(t\right)=s_{3}\left(t\right)-s_{4}\left(t\right). \end{array}$$

The adjusting ISI (*I*_*a*_(*t*)) (Figures 1C,D), which is the basis for all four methods, is defined as

(3) $$\begin{array}{l} I_{a}(t)=\{\begin{array}{ll} i_{1}(t), & \text{if }t-s_{1}(t)<i_{1}(t)\\ t-s_{1}(t), & \text{otherwise} \end{array}. \end{array}$$

The adjusting ISI increases linearly if the difference of the actual time *t* to the previous spike (*s*_1_(*t*)) is greater than the previous ISI (*i*_1_(*t*)). This case occurs in Figure 1C e.g., around 1 s. The advantages of using this definition of adjusting ISI are (1) no knowledge of the future spike times is needed and (2) the adjusting ISI increases automatically if the last ISI (*i*_1_(*t*)) is shorter than the period between the actual time point to the last spike (*t* − *s*_1_(*t*)).

Two ISI-methods (ISI-Ratio and classification) require additional knowledge of previous ISIs for comparison with the adjusting ISI. For this purpose a weighted mean of the two previous ISIs (*I*_*pre*_(*t, ω*)) was employed. We consider relative weights of the second last ISI and the last ISI in the range from *ω* = 0 (no effect of the second last ISI, only of the last ISI), via *ω* = 0.5 (same effects of last and second last ISI), to *ω* = 1 (no effect of the last ISI, only of the second last ISI). Consequently, the weighted previous ISI is defined as

(4) $$\begin{array}{l} I_{pre}(t,\omega )=\{\begin{array}{l} \left((1-\omega )\cdot i_{1}(t)+\omega \cdot i_{2}(t)),\text{if}t\neq s_{1}(t\right)\\ \left((1-\omega )\cdot i_{2}(t)+\omega \cdot i_{3}(t)),\text{if}t=s_{1}(t\right) \end{array},\;\omega \in [0,1]. \end{array}$$

If only one previous ISI was considered, *ω* was equal to 0 $\left(I_{pre}\left(t,\text{0}\right)=\{\begin{array}{cc} i_{1}(t), & \text{if }t\neq s_{1}(t)\\ i_{2}(t), & \text{if }t=s_{1}(t) \end{array}\right).$

Consequently, *I*_*pre*_(*t*, 0) is in many time points *t* identical to the adjusting ISI *I*_*a*_(*t*), which is illustrated in Figures 1C, 2B. The two curves only differ when a new spike is generated or when the adjusting ISI increases linearly. An example of the weighted previous ISI with a weight of 0.5 (*I*_*pre*_(*t*, 0.5)) is shown in Figure 2C1.

### 2.3. Stimulus Change Point Detection Methods

#### 2.3.1. Moving-Average Method

The Moving-Average method was selected for comparison with our proposed ISI-methods as the standard approach that is most frequently found in neuroscientific studies. In this method (see also Koepcke et al., 2016) the actual spike rate was compared to a mean spike rate and a multiple of the standard deviation based on the previous recording period. To keep the required previous recording as short as possible, we used the instantaneous spike rate as an estimate of the spike rate at each time point.

The instantaneous spike rate (ISR) is defined as the inverse of the adjusting ISI (Equation 3):

(5) $$\begin{array}{l} \hat{r}\left(t\right)=\frac{1}{I_{a}\left(t\right)}. \end{array}$$

By applying the adjusting ISI, this method is a true online method, that does not employ any future information. However, it differs slightly from published ISR definitions (Pauluis and Baker, 2000; Lánský et al., 2004; Kostal et al., 2018), which rely the next spike to determine the ISI and its corresponding inverse. Because in our definition the ISR is the reciprocal of *I*_*a*_(*t*), the estimated spike rate of the cell decreases hyperbolically when the adjusting ISI increases until the next spike is generated (Figure 2A1).

The mean ($\bar{r}$) of the ISR and its standard deviation (*s*) is calculated for every point *t* in the interval [*t* − *W, t*] of length *W* (Figure 2A2). We tested 17 different window sizes (*W* = 5, 10, …, 50, 60, 70, …, 100, 150, 200 ms). Threshold crossings are:

(6) $$\begin{array}{l} E_{in}(t)=\{\begin{array}{c} \begin{array}{c} 1,\text{ if }\hat{r}(t)>\bar{r}(t)+\theta_{in}s(t)\\ 0 \end{array} \end{array},\\ E_{de}(t)=\{\begin{array}{c} 1,\text{ if }\hat{r}(t)<\bar{r}(t)-\theta_{de}s(t)\\ 0 \end{array}. \end{array}$$

*θ*_*in*_, *θ*_*de*_ > 0 denote the units of standard deviation used as the thresholds for CP detection. The suffixes “in” and “de” indicate stimulus in- and decrease, respectively, used for the two different thresholds required for asymmetric distributions. The threshold parameter spaces covered by 300 tested values are listed in Table S2. Since only the first time point *t* after a threshold crossing should be considered as a putative CP, but not the succeeding time points while the threshold stays crossed, putative CPs are defined as:

(7) $$\begin{array}{l} {\text{CP}}_{in}=\{t\;|\;E_{in}(t)=1\wedge \forall \tau \in [s_{1}(t),t[:E_{in}(\tau )=0\}, \end{array}$$

(8) $$\begin{array}{l} {\text{CP}}_{de}=\{t\;|\;E_{de}(t)=1\wedge \forall \tau \in [s_{1}(t),t[:E_{de}(\tau )=0\}. \end{array}$$

Please note that CP_*in*_ can only occur at spike times *t*_*j*_, while *θ*_*de*_ is usually crossed between spike times.

#### 2.3.2. Pure-ISI Method

We introduce the Pure-ISI method as the simplest CP detection method a neuron could apply. The only information it uses is the actual neuronal activity with the underlying assumption that a particularly short or long ISI indicates a stimulus change. Therefore, this methods tries to separate the distribution of the adjusting ISI into the three conditions by applying two thresholds (*θ*_*in*_ and *θ*_*de*_) for finding particularly short or long adjusting ISIs (section 2.2.2, Figure 1C) to detect putative CPs (Figures 2B1–B3). The putative CPs are:

(9) $$\begin{array}{l} {\text{CP}}_{in}=\{t\;|\;I_{a}(t)<\theta_{in}\wedge \forall \tau \in [s_{1}(t),t[:I_{a}(\tau )\geq \theta_{in}\},\;\theta_{in}>0, \end{array}$$

(10) $$\begin{array}{l} {\text{CP}}_{de}=\{t\;|\;I_{a}(t)>\theta_{de}\wedge \forall \tau \in [s_{1}(t),t[:I_{a}(\tau )\leq \theta_{de}\},\;\theta_{de}>0 \end{array}$$

with *θ* denoting a pre-defined threshold for detecting changes and the suffixes “in” and “de” again indicating stimulus increase and decrease, respectively. The threshold parameter spaces sampled by 600 tested values are listed in Table S2. Again, CP_*in*_ always refer to spike times *t*_*j*_, while CP_*de*_ are usually between spike times.

#### 2.3.3. ISI-Ratio Method

As a more elaborate, but still biologically plausible CP detection method, we introduce the ISI-Ratio method, which compares the adjusting ISI (*I*_*a*_(*t*)) with the weighted previous ISI (*I*_*pre*_(*t, ω*)) by analyzing their ratio (*R*(*t, ω*)) (section 2.2.2, Figure 2C2).

(11) $$\begin{array}{l} R\left(t,\omega \right)=\frac{I_{a}\left(t\right)}{I_{pre}\left(t,\omega \right)}. \end{array}$$

The bigger *ω* the stronger is the impact of the second last ISI on the ISI-Ratio. The effect of this weight was analyzed with 17 different weights (ω = [0, 0.0625, 0.125, …, 1]). An example of the adjusting and previous ISI is shown in Figures 2C1,D1 and the ratio in Figure 2C2.

For intensity increases a putative CP is detected at a spike time *t*_*j*_ if the ratio is smaller than a threshold *θ*_*in*_ < 1 under the condition that the ratio at the previous spike time (*t*_*j*−1_) did not reach the threshold (Figure 2B2). For detecting intensity decreases the ratio has to be greater than a threshold *θ*_*de*_ > 1 at any time point *t* and the ratio has to be smaller at the last spike time *s*_1_(*t*). Therefore, putative CPs are defined as

(12) $$\begin{array}{l} {\text{CP}}_{in}=\{t\;|\;R(t,\omega )<\theta_{in}\wedge \forall \tau \in [s_{1}(t),t[:R(\tau ,\omega )\geq \theta_{in}\}, \end{array}$$

(13) $$\begin{array}{l} {\text{CP}}_{de}\;=\{t\;|\;R(t,\omega )>\theta_{de}\wedge \forall \tau \in [s_{1}(t),t[:R(\tau ,\omega )\leq \theta_{de}\}. \end{array}$$

The 500 tested values for *θ*_*in*_ and *θ*_*de*_ are listed in the Table S2.

#### 2.3.4. Classification-Method

**Figure 3:**
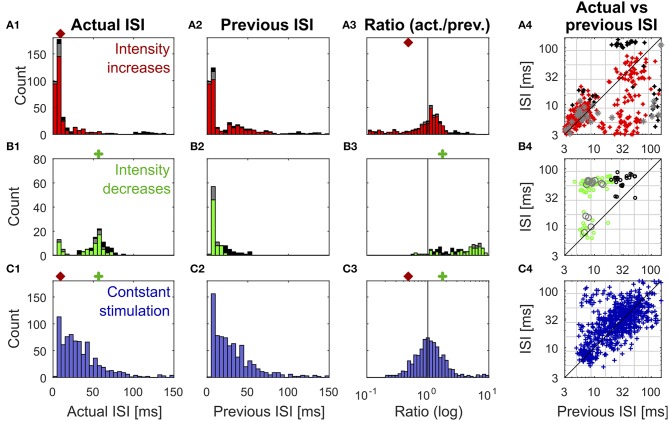
Distributions of previous and actual ISI and their relationship under three different conditions. The data contains all ISIs in 10 recorded trials of the experiment shown in Figure 1. The data is divided according to the stimulation that elicited the analyzed spikes into responses to stimulus intensity increases **(A1–A4)** (red: IFB, gray: IBB, black: onset), intensity decreases **(B1–B4)** (green: DBB, gray: DFB, black: offset), and constant simulation **(C1–C4)** (blue). The data of the intensity in- and decreases were taken from the accepted time ranges (section 2.4, Figure 1C). The first column **(A1–C1)** shows the histograms of the actual ISI for all three conditions (Figure 1D). The red and green markers represent the example thresholds, which were applied in Figure 2B by the Pure-ISI method. The second column **(A2–C2)** illustrates the distributions of the previous ISI (*i*_1_, section 2.2.2; Figure 1D). The third column **(A3–C3)** shows the ISI-Ratios of the previous and actual ISI ($\frac{\text{actual ISI}}{\text{previous ISI}}$). The scale of the x-axis is logarithmic. The red and green markers indicate the example thresholds used for the ISI-Ratio in Figure 2C. The scatter plots in the right column **(A4–C4)** show the pair of previous and actual ISI for each spike on a logarithmic scale. The colors again indicate the type of in- or decrease for each data point.

For rigorous evaluation of our introduced ISI-methods, we chose to use a naive Bayes classifier, which is frequently applied in machine learning (Hand and Yu, 2001). This method should act as reference for an upper limit of the CP detection performance that can be obtained for the given data. In contrast to the ISI-Ratio method, the classification method makes use of the absolute values of the ISIs (Figures 3A–C4). The main idea is to use a training data set to learn the statistical properties of the ISIs during constant stimulation and in response to stimulus changes. The method was applied for the detection of in- and decreased activity changes independently.

Applying leave-one-out cross-validation, one training data set consisted of the trials *l* = [1, …, *m* − 1] and the test data set the *m*-th trial. (In our specific case, *m* = 10 trials were used.) Here, for simplification of the description only the case of intensity increases is illustrated, decreases were identified equivalently.

First the adjusting and weighted previous ISI (*I*_*a*_(*t*), Equation (3), *I*_*pre*_(*t*, ω), Equation (4)) were calculated for all training trials *l*. The next step was to replace in every time point the continuous values of the adjusting and previous ISI by discrete categories (Figures 3A–C4, 2D1). The discrete categories were determined by the logarithmic spacing between the minimum and the maximum ISI of the training data set. In this study, we divided the interval into *k* = 10 categories.

(14) $$\begin{array}{l} C_{a}\left(t,l\right)=c,\;\text{for}e_{c-1}<I_{a}\left(t\right)\leq e_{c},\\ C_{pre}\left(t,l,\omega \right)=c,\;\text{for}e_{c-1}<I_{pre}\left(t,\omega \right)\leq e_{c}. \end{array}$$

The borders [*e*_0_, …, *e*_*k*_] of the categories were defined as:

(15) $$\begin{array}{l} e_{0}=0,\;e_{1}=a{\left(ba^{-1}\right)}^{\frac{1}{k}},\;e_{2}=a{\left(ba^{-1}\right)}^{\frac{2}{k}},\ldots ,\\ e_{k-1}=a{\left(ba^{-1}\right)}^{\frac{k-1}{k}},e_{k}=\infty , \end{array}$$

where *a* and *b* are the minimal and maximum ISI, respectively, which occurred in the training data set. For comparing the category of the adjusting ISI at time point *t* and trial *l* with the category of the weighted previous ISI both categories were combined:

(16) $$\begin{array}{l} C_{pair}\left(t,l,\omega \right)=\left(C_{pre}\left(t,l,\omega \right),C_{a}\left(t,l\right)\right). \end{array}$$

In total, *k*^2^ possible combinations exist. The categories *C*_*pair*_(*t, l*, ω) were further subdivided into two groups: one group corresponding to the activity in response to an intensity increase (*T*_in_), and the other group covering constant stimulation or intensity decreases (*T*_c,de_). The determination of *T*_in_ depends on the response latency of the system. We set *T*_in_ equal to the accepted time range, in which a putative CP was classified as true positive (section 2.2.1).

(17) $$\begin{array}{l} N_{\text{in}}\left(k_{1},k_{2}\right)=\#\left\{t\;|\;C_{pair}\left(t,l,\omega \right)=\left(k_{1},k_{2}\right),t\in T_{\text{in}}\right\}, \end{array}$$

(18) $$\begin{array}{l} N_{\text{c,de}}\left(k_{1},k_{2}\right)=\#\left\{t\;|\;C_{pair}\left(t,l,\omega \right)=\left(k_{1},k_{2}\right),t\in T_{\text{c,de}}\right\}. \end{array}$$

The relative frequency for every category combination was calculated:

(19) $$\begin{array}{l} f\left(k_{1},k_{2}\right)=\frac{N_{\text{in}}\left(k_{1},k_{2}\right)}{N_{\text{c,de}}\left(k_{1},k_{2}\right)+N_{\text{in}}\left(k_{1},k_{2}\right)}. \end{array}$$

For the test data set (trial *m*), the adjusting and previous ISI, as well as their categories were calculated. With the combined categories, a CP probability (*P*) with the relative frequencies of Equation (19) was determined (Figure 2D2)

(20) $$\begin{array}{l} P\left(t\right)=f\left(C_{pre}\left(t,m,\omega \right),C_{a}\left(t,m\right)\right). \end{array}$$

Threshold crossings occur if the probability of a stimulus change was greater than a pre-defined threshold *θ*. Then are the putative CPs:

(21) $$\begin{array}{l} \text{CP}=\{t\;|\;P(t)>\theta \wedge \forall \tau \in [s_{1}(t),t[:P(\tau )\leq \theta \},\;\theta \in [0,1]. \end{array}$$

For the weighted previous ISI we tested the same 17 different weights between 0 and 1 as for the ISI-Ratio method. The 200 tested values of *θ* are given for intensity in- and decreases in Table S2.

### 2.4. Evaluation Technique: ROC Curve and AUC-Value

In a receiver operating characteristic curve (ROC curve), the true positive rate (TP-Rate) is plotted against the false positive rate (FP-Rate; Krzanowski and Hand, 2009; Aminikhanghahi and Cook, 2017). One point on the ROC curve (FP-Rate, TP-Rate) corresponds to one parameter setting of a method (Figures 2A–D3). To evaluate a CP method the Area Under the Curve (AUC) was calculated (Aminikhanghahi and Cook, 2017). It can be interpreted as the probability increase of detecting a time point after a stimulus change compared to a randomly chosen time point during constant stimulation (Hanley and McNeil, 1982).

The evaluation technique is described here based on the intensity increases, the intensity decreases were evaluated analogously. Because the recordings are continuous time series, a modification of the TP- and FP-Rate was necessary. The TP-Rate is the number of TP (#TP) divided by the number of intensity increases (*n*_*in*_). The FP-Rate is the ratio between the number of FPs (#FP) and a number of intervals where constant stimulation or intensity decreases are present. Each intensity increase refers to an interval of the accepted time range (*T*_accept_), where a CP is identified as true positive. The rest of the recording duration contains neuronal activity which is not triggered by an intensity increase. The FP-Rate is normalized by the maximum number of CPs that could theoretically be reached during the recording, calculated as the ratio of the total recording duration *D* and the length of the accepted time range |*T*_accept_|, from which the number of intensity increases (*n*_*in*_) is subtracted.

(22) $$\begin{array}{l} \text{TP-Rate}=\frac{\text{\#TP}}{n_{\text{in}}};\;\text{FP-Rate}=\frac{\#\text{FP}}{D/|T_{\text{accept}}|-n_{\text{in}}}. \end{array}$$

In theory, the FP-rate can be greater than 1, but in practice, the maximal FP-rate was normally around 1. For calculating the AUC-value the TP-Rate and FP-Rate were determined for each trial independently and the mean was analyzed.

## 3. Results

The goal of this study is to test two simple, biologically realistic approaches for identifying multiple stimulus changes in neuronal responses. These methods are compared to two established change point detection methods by applying them to intracellularly recorded responses to acoustical stimulation of the cricket's AN2 neuron. The method's robustness was checked with extracellularly recorded spike trains elicited by the same stimulation of 16 kHz, as well as with responses to 3 kHz stimulation. The stimulation protocols comprised three different types of intensity in- and decreases (section 2.1, Figure 1A): sound onset (onset), increase from baseline (IFB) and back to baseline (IBB); sound offset (offset), decrease from baseline (DFB) and back to baseline (DBB).

### 3.1. ISI and ISI-Ratio Distributions

All methods rely on the hypothesis that the distributions of “actual” ISI and its previous ISI are distinguishable under the three conditions (intensity in- and decreases and constant stimulation). It is obvious that the histograms of the actual ISI and the ISI-Ratio (actual ISI/previous ISI) differ between the different conditions (Figure 3). However, they also overlap, in particular for the intensity decreases and constant stimulation.

Figure 3 shows the distributions of the actual ISI (Figures 3A–C1), its previous ISI (Figures 3A–C2) and their relationship in form of the distribution of their ratio (Figures 3A–C3) and a scatterplot of their combination (Figures 3A–C4) for one example experiment. The distributions are displayed in form of histograms under the three conditions (intensity increase; Figures 3A1–A4) and decreases (Figures 3B1–B4), constant stimulation (Figures 3C1–C4). The displayed data for the actual ISI correspond to all ISIs (*i*_1_, Equation 2) recorded within the accepted time range (*T*_true_) after each intensity change (section 2.2.1). Hence, the individual stimulus periods contribute different amounts of data points, depending on the respective spike rates they triggered. The colors in each of the distributions and scatter plots indicate the different sub-types of intensity in- and decreases. They show that positive sound intensity steps leading to IFBs (red) and DBBs (green) occurred more often in the data set than gaps leading to DFBs and IBBs (gray), as well as stimulus on- and offsets (black).

Moreover, the adjusting ISI of the end of the respective accepted time ranges was added to the displayed data, which is crucial for the detection of reduced neuronal activity during intensity decreases. A pause in the spiking activity as a response to an intensity decrease lasted usually longer than the accepted time range (Figure 1). Hence, this long ISI needs to be considered at the end of the accepted time range to prevent it from being falsely assigned to the succeeding baseline stimulation.

#### 3.1.1. Intensity Increases

ISIs (Figures 3A–C1) and ISI-Ratios (Figures 3A–C3) tended to be smaller for the intensity increases compared to the other two conditions, even though the distributions overlap. However, for the definition of strong responses only the shortest ISI occurring during the stimulation had to be lower than the threshold of 6.5 ms (typical values were 2.5–4 ms) and the smallest ISI-Ratio had to be smaller than 0.2. According to this definition, strong responses occurred in ~70% of the intensity increases. They were elicited by most of the IFBs (red in Figure 3) and onsets (black) as well as by a quarter of the IBBs (gray). In 12% of the intensity increases (mainly IBBs) no increase of the neuronal activity was measurable (ratio > 1 in all time bins) and the corresponding minimum ISI was quite long (> 40 ms).

In addition to the percentages of strong responses, the latency between an intensity increase and the occurrence of the minimum ISI differed between the intensity increase types. On average, the minimum ISI was found earlier for IFBs than for onsets and IBBs. For half of the intensity increases, the spike time with the minimum ISI-Ratio coincided with the spike time of the minimum ISI. In the other cases, mainly for IFBs, the minimum ISI occurred one spike later than the minimum ISI-Ratio.

#### 3.1.2. Intensity Decreases

In contrast to the intensity increases, far fewer spikes occurred after intensity decreases. This is illustrated by the size of the histograms of the actual ISI and ISI-Ratio in Figures 3B1,B3. For most intensity decreases, the ISI-Ratio was between 2 and 6, clearly indicating decreased neuronal activity. Only in 4% of all intensity decreases no decrease of the neuronal activity (ISI-Ratio < 1 in all time bins) was measurable. For DFBs the ISIs tended to be even longer. But the ratios within the time range were smaller than for the other intensity decrease types.

Since the detection of intensity decreases relies on the maximum adjusting ISI and ISI-Ratio, detection performance depends critically on the definition of the accepted range. For the standard experiments (16 kHz stimulation, intracellular recording), the typical range found for the maximum ISI was between 38 and 51 ms. The fact that 55–75% of the maximum ISI and ISI-Ratio were found at the end of the accepted interval of 15–55 ms indicates that the response lasted longer than 55 ms. Hence, the choice of a longer accepted time range would result in higher ISIs and ISI-Ratios, but would not be appropriate for the given stimulation protocol (refer to the Supplementary Material for the definition of the accepted time range).

### 3.2. Comparison of CP Detection Methods

**Figure 4:**
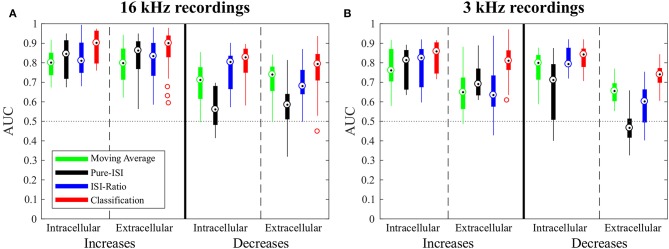
Overview of change point detection performances. The boxplots contain the best AUC-values of every experiment for each method. For parameter optimization, the weight of the second last ISI influencing the ISI-Ratio and classification method was limited to the range between 0 and 0.5 and the window size of the Moving-Average method was limited to a maximum of 100 ms. **(A)** Responses to 16 kHz stimulation: intracellular recordings (*n* = 9) and extracellular recordings (*n* = 43); **(B)** Responses to 3 kHz stimulation: intracellular recordings (*n* = 11) and extracellular recordings (*n* = 30).

**Figure 5:**
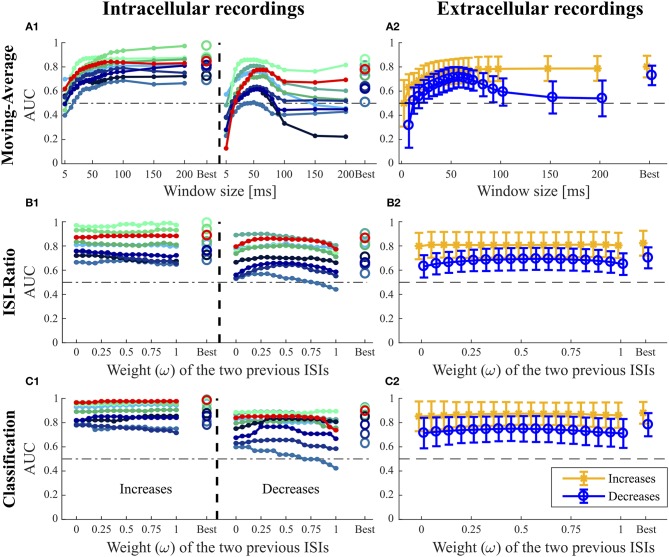
Influence of the additional parameters on the AUC-values for the Moving-Average **(A1,A2)**, ISI-Ratio **(B1,B2)** and classification method **(C1,C2)**. **(A1,B1,C1)** For the nine intracellular recordings of responses to 16 kHz stimulation, the AUC-values are illustrated separately with the red line representing the performance of the example data set in Figures 1–3. **(A2,B2,C2)** The performances obtained for the 43 extracellular recordings are summarized by means (circles) and standard deviations (error bars) over experiments. For the Moving-Average method **(A1,A2)**, the influence of the moving average window size on the AUC-value is shown. For the ISI-Ratio **(B1,B2)** and **(C1,C2)** classification, the weight of the two previous ISIs was varied to analyze their influence on the AUC-value. 17 weights between 0 and 1 were tested for calculating the previous ISI (*I*_*pre*_(*t*, ω), Equation 4). A weight of 0 indicates no influence of the second last ISI, whereas the last ISI had no influence if the weight was equal to 1. For the corresponding analysis performed on the responses to 3 kHz stimulation, please refer to Figure S1.

In this section, the four CP detection methods Moving-Average, Pure-ISI, ISI-Ratio, and classification are compared based on the standard data set, intracellular recordings of responses to 16 kHz stimulation. The overall comparison of detection performances for intensity in- and decreases is illustrated in Figure 4, before analyzing the effects of parameter variations for the four methods individually (Figure 5). The AUC-values shown in the boxplots in Figure 4 result from the combined performances of the different types of in- and decreases. For the intensity increases all methods yielded similarly good results. For the standard experiments, the widely used Moving-Average achieved AUC-values between 0.75 and 0.85 (Figure 4A, green). The simpler methods Pure-ISI (black) and ISI-Ratio (blue), which rely only on one or two ISIs for stimulus change detection, performed similarly well or even slightly better than the Moving-Average method. The classification method with a median AUC-value of 0.9 was very successful in detecting increases in stimulus intensity. Altogether all methods were suitable for detecting stimulus intensity increases in the standard experiments.

For detecting intensity decreases, greater performance differences were found between the methods. The Moving-Average method was less successful than for detecting intensity increases. The AUC-values were on average 0.12 lower, resulting in AUC-values between 0.6 and 0.77 for the standard experiments. The ISI-Ratio and classification methods also achieved lower AUC-values for the detection of intensity decreases, but the differences in performance were less pronounced than for the Moving-Average method. The Pure-ISI method showed a drastically lower performance. For many experiments this method failed to identify intensity decreases which lead to very low AUC-values (median 0.54). Hence, the ISI-Ratio method and the classification method were suitable to detect stimulus intensity decreases, whereas the Moving-Average achieved mixed results and the Pure-ISI method failed for this detection task.

#### 3.2.1. Moving-Average Method

Our main findings for the Moving-Average method (section 2.3.1) were that it was suitable to detect intensity increases, especially when they caused strong responses, but it had some difficulties in detecting intensity decreases. The performance strongly depended on a stable activity in a long reference period.

Figures 2A1–A3 shows the results of the Moving-Average method for the example experiment. The dark dotted line in Figure 2A2 displays the Moving-Average of the ISR calculated with a window size of 70 ms. The gray area marks the mean plus/minus a multiple of the standard deviation. A CP was detected when the ISR exceeded or fell below the gray area.

##### 3.2.1.1. Intensity increases

The Moving-Average method was found to be well-suited for the detection of intensity increases (Figures 4, 5A1,A2). The size of the moving average window had a strong influence on the CP detection performance (Figures 5A1,A2), With increasing window size, the reliability of detection rose and AUC values increased visibly up to a saturation value at ~80 ms. For the comparison of methods' performances in Figure 4, the window size was restricted to 100 ms. Longer windows for averaging would not be biologically plausible and could cause a strong influence of previous stimulus changes on the reference activity. Under this restriction, the optimal window size was found to be between 80 and 100 ms for most of the experiments. If this restriction was abolished, AUC values increased with window sizes of up to 200 ms for many of the experiments (Figures 5A1,A2).

The Moving-Average method is generally suitable to detect intensity increases. Good performances were obtained in particular for strong responses. Medium responses (shortest ISIs 7–40 ms and smallest ISI-Ratio 0.2–0.5) still lead to acceptable performances, while the method mostly failed in detecting weak responses. The example experiment shown in Figure 2 yielded a good AUC-value of 0.87. The corresponding ROC-curve in Figure 2A3 (green line) illustrates the dependency of the TP-Rate on the FP-Rate. The strong responses elicited by onsets and IFBs led to high detection rates (90% for a FP-Rate of 0.05, Figure 2A2). Also half of the IBBs were detected in this example. However, there was some variability between the data sets. For some recordings, the Moving-Average method could detect only half of the IFBs when the FP-Rate was fixed to 0.05.

##### 3.2.1.2. Intensity decreases

For detecting intensity decreases, the window size had an even more severe impact on the performance (Figures 5A1,A2), because the Moving-Average method requires stable reference data. The AUC-values increased up to a window size of 50–70 ms and then decreased again. The reason for this optimum window size was the design of the stimulus protocol, in which the duration between an IFB and the following DBB was 50 ms. Adding the corresponding response latency, we obtained a relatively stable interval of 50–70 ms. For DFBs longer reference windows were better suited because the last intensity change was more than 200 ms ago (section 2.1, Figure 1). Hence, long constant reference activity improved the performance.

Even with optimized window sizes the Moving-Average method had problems to detect intensity decreases occurring after a strong response to an intensity increase (mostly DBBs). This is reflected in low TP-Rates of around 0.3 (median) with a fixed FP-Rate of 0.15. For example, in the example recording shown in Figure 2A3 42% of all DBBs were detected. The resulting AUC-value of 0.68 indicates the low performance for detecting decreases. Hence, the Moving-Average method was not a good choice for the given data set, containing low spike rates and short periods of stable activity due to the specific stimulus time course.

#### 3.2.2. Pure-ISI Method

The Pure-ISI method (section 2.3.2) is the simplest of the methods tested in this study, because it relies exclusively on one adjusting ISI. The main results obtained for this method are that it could reliably detect strong responses, but had problems detecting responses with longer ISI, in particular for IBBs. Intensity decreases were only rarely found within the accepted time range, particularly for decreases after a strong response (offsets and DBB). For given FP-Rates, the thresholds for detection of activity in- and decrease depended on the average spike rates.

Figure 2B2 shows an example application of the Pure-ISI method, in which a putative CP was detected, when an ISI was shorter than *θ*_*in*_ = 9.8 ms (detection of intensity increases) or longer than *θ*_*de*_ = 57 ms (detection of intensity decreases). These thresholds (also marked in the ISI distributions in Figures 3A–C1) correspond to FP-Rates of 0.05 for stimulus increases and 0.15 for decreases.

We varied the two thresholds systematically to calculate the TP- and FP-Rates for the ROC curve. The thresholds leading to given FP-Rates (0.05 for stimulus increases and 0.15 for decreases) depended on the neuronal activity. The thresholds for intensity increases (*θ*_in_) and intensity decreases *θ*_in_ correlated with the average ISI following intensity changes (correlation coefficient of 0.7 for increases and 0.75 for decreases). The threshold ISI for intensity increases (*θ*_in_) was ~1–2 ms shorter and for intensity decreases (*θ*_de_) ~20 ms longer than the average ISI, both of which were significant differences (paired Wilcoxon-rank-sum-test, *p* < 0.05).

##### 3.2.2.1. Intensity increases

The successful application of the Pure-ISI method for detecting strong responses to stimulus intensity increases can be observed for the example data set in Figure 2B. The reliable detection of strong responses resulted in a high TP-Rate for onsets and the most frequently occurring IFBs. However, this method had problems to detect medium and weak responses to intensity increases. Only 30% of the less frequently occurring IBBs were detected in the example of Figure 2B, still leading in total to a high AUC-value of 0.9, which is in the upper range of the AUC-values obtained for the detection of stimulus increases with this method (Figure 4A).

##### 3.2.2.2. Intensity decreases

Considerably lower performance values were obtained for detecting intensity decreases compared to increases. Since the adjusting ISI after a DFB was generally longer than the ISIs during constant stimulation, these CPs could be detected by the Pure-ISI method. In contrast, the adjusting ISIs within the accepted range after a DBB or offset were often shorter than ISIs during constant stimulation, which lead to a low detection performance. For the example in Figure 2B2, none of the DBBs and 40% DFBs were detected, causing a low AUC-value of 0.6. The detected responses had long adjusting ISIs (> 57 ms).

The poor performances for detecting DBBs and offsets were reflected in the AUC-values (Figure 4A). Some data sets even yielded AUC-values below 0.5, which is worse than flipping a coin. If the first ISI after an intensity decrease was short, false positives (indicating putative stimulus increase) were detected before true positives (indicating stimulus decrease) could be found. This problem concerned the Pure-ISI method and the classification more strongly than the ISI-Ratio and Moving-Average, in which relative activity changes rather than crossings of absolute ISI values were used for detection.

#### 3.2.3. ISI-Ratio Method

The ISI-Ratio method (section 2.3.3) was found to be a very good choice for detecting both intensity in- and decreases. For decreases the performance was improved by taking the second last ISI into consideration for the ratio.

The ISI-Ratio method assumes that changes in the stimulus are represented in the weighted ratio of the adjusting and the previous ISI (e.g., Figures 2C1,C2). The method subdivides the distribution of ratios into three parts by applying one threshold for increases and another for decreases (Figures 3A–C3) with example thresholds *θ*_*in*_ = 0.48, *θ*_*de*_ = 1.84). The same thresholds were also applied in Figure 2C2, which shows an example of the ISI-Ratio method with a weight of *ω* = 0 in detecting increases with a FP-Rate of 0.05 and decreases with a FP-Rate of 0.15.

##### 3.2.3.1. Intensity increases

In Figures 5B1,B2, the effect of the weight ω ∈ [0, 1] (Equation 4) on the AUC-values is analyzed with higher weights referring to a stronger influence of the second last ISI on the ratio. The curves of the example recording (red line) and other experiments in Figure 5B1 indicate that the weight *ω* did not affect the AUC-value for intensity increases. Hence, considering the second last ISI neither deteriorated nor improved the performance compared to using only the last ISI for CP detection.

Stimulus intensity increases triggering very short ISIs also caused a low ISI-Ratio (< 0.2) for the first ISI after stimulus transition. These strong responses were reliably detected by the ISI-Ratio method (Figure 2C2). Since the method also showed acceptable performances for medium responses, good total AUC-values were obtained for intensity increases (Figure 4).

##### 3.2.3.2. Intensity decreases

In contrast to the intensity increases, the weight *ω* had a noticeable effect on the AUC-value for intensity decreases (Figures 5B1,B2). Best AUC-values were obtained for weights between 0.25 and 0.75, which correspond to an approximately equal influence of both previous ISIs. Maximum AUC-values were reached for weights close to 0.5 with a median improvement of 0.055 units compared to the AUC-value obtained when the second previous ISI was not taken into account. Especially the detection of DBBs, which occurred after a strong response to IFBs, benefited from taking the second last ISI into consideration.

Additionally, the ISI weight influenced the threshold level, which in turn effected the detection times. When the FP-Rate was fixed to 0.15, weights between 0.25 and 0.75 offsets and DBBs were detected 1–2 ms and DFBs even 8 ms earlier than when only the last ISI was used for CP detection.

When optimizing the weight relative to the AUC-values for direct comparison of the four methods (Figure 4), *ω* was restricted to the interval between 0 and 0.5, because a stronger effect of the second last compared to the last ISI would be biologically implausible. The ISI-Ratio method showed good detection performance for responses with a ISI-Ratio greater than 1.5, which mostly occurred for DBBs after a strong response to IFB. For the standard experiments (Figure 2C2) almost all offsets and DBBs and 25% of the DFBs could be detected corresponding to a total median TP-Rate of 65%. In total, all intracellular recordings stimulated with 16 KHz yielded a median performance of 0.8 for the detection of intensity decreases (Figure 4).

#### 3.2.4. Classification Method

The classification (section 2.3.4) considering combinations of ISI distributions (Figures 3A–C4) is the most complex method tested in this study. As expected, it yielded a high detection rate for stimulus intensity in- and decreases (Figure 4).

The different distributions triggered by the three different conditions suggest that they could be used as the basis for the discrimination of stimulus changes from constant stimulation. However, the still substantial overlap in the distributions caused a problem for applying the classification method to our data set. Some stimulus changes did not trigger strong changes in activity. Since these responses were part of the training data, the classification method learned that constant activity could indicate a stimulus change. This “false” training was mainly visible for detecting stimulus intensity decreases. It lead to a large fraction of false positives, because putative CPs were detected shortly after or even before the stimulus changed. On the other hand, the “false” training also lead to the correct detection of stimulus changes in some cases, in which spikes were suppressed by stimulus decreases.

In Figures 2D1–D3, classification of the example experiment was performed with equally strong influence of both previous ISI (ω = 0.5). Figure 2D2 illustrates the estimated probabilities for intensity in- and decreases based on the training data set (consisting of the other 9 trials of this experiment) and on a specific parameter combination. The training of increase detection was based on the interval (*T*_true_ = [10, 40] ms) after each stimulus intensity increase. In contrast, the interval to learn the statistics of responses to stimulus decreases was shifted to a later starting point ([20,65] ms) to reduce the effect of “false” training, which nevertheless occurred to some extent. For this example, the resulting probability thresholds determined by the training were 0.45 for intensity increases and 0.2 for decreases (Equation 21), reflecting the broadly overlapping distributions obtained for intensity decreases and constant stimulation (Figures 3B–C4).

##### 3.2.4.1. Intensity increases

When varying the weight of the two previous ISIs, no general effect on the AUC-value could be observed for the detection of intensity increases (Figures 5C1,C2). The second last ISI did not influence the performance in most of the experiments, in some experiments it even had a slightly negative effect. Like for the ISI-Ratio method, the weight was optimized relative to the AUC-values and restricted to the range between 0 and 0.5 for the sake of biological plausibility in methods comparison (Figure 4). Generally, the classification method achieved a very good median AUC-value of 0.9. Similar to the other methods, good results were generally obtained for the detection of strong responses, while the performance for medium responses was slightly reduced. In contrast to the other approaches, the classification method also detected many weak responses. However, these true positives were the result of the “false” training effect as becomes evident from the detection times. While neuronal responses usually occur the earlier the stronger they are, the classification method often identified the absence of a neuronal response as indication of a putative CP very shortly after the stimulus changed.

This effect is also visible for the example experiment (Figures 2D1–D3), which achieved a high total AUC-value of 0.95. In addition to the detection of all onsets and IFBs, which caused strong responses, also 80% of the IBBs were detected. Due to the “false” training, the median detection time of the weak or missing responses to IBBs was with 18 ms even shorter than the median detection time of 20 ms for the vigorous responses to IFBs.

##### 3.2.4.2. Intensity decreases

In contrast to the finding for intensity increases, the weight of the previous ISI effected the AUC-value obtained for the detection of intensity decreases more clearly (Figure 5C1). Similarly to the ISI-Ratio method, best results were obtained for weights around *ω* = 0.5. Here, the reason for this effect was a better separation of the dots within the logarithmically spaced categories for weights between 0.25 and 0.625. Therefore, more intensity decreases could be detected with a lower FP-Rate.

The classification method yielded a total median AUC-value of ~0.8 for stimulus intensity decreases. Examining the different stimulus types, this method was well-suited to detect stimulus intensity decreases after a period of strong neuronal response (DBBs). Intensity decreases from baseline (DFBs) were not detected as well.

For the example experiment (Figure 2D2), 78% of all decreases could be identified. This number resulted from the classification method detecting all offsets and DBBs, but only 15% of DFBs. The scatter plot in Figure 3B4 indicates the substantial overlap with the values found during constant stimulation (Figure 3C4), leading to false negatives. Still, the classification method gained for this example a total AUC-value of 0.8.

### 3.3. Additional Data Sets

#### 3.3.1. 3 kHz Intracellular Experiments

Compared to the standard experiments, the 3 kHz intracellular experiments showed much more variability in the response behavior to stimulus intensity changes. Only 45% of the intensity increases triggered a strong response (shortest ISI <6.5 ms). In particular, onsets and IBBs elicited lower numbers of strong responses than were observed for 16 kHz stimulation. Nevertheless, the percentage of stimulus intensity increases that failed to induce a neuronal activity increase (ISI-Ratio >1, ISI >40 ms) was approximately the same for stimulation with 16 kHz and with 3 kHz.

When applied to intensity increases, all methods achieved similar or slightly lower AUC-values for responses to 3 kHz stimulation than for the standard experiments (Figure 4). Moreover, the same trends were visible for the influence of the additional parameter on the AUC-values (Figure 5 and Figure S1).

For the detection of intensity decreases, the comparison of 3 and 16 kHz lead to different results depending on the applied method. Similar performances were observed for intracellular experiments stimulated with both frequencies when applying the ISI-Ratio or the classification method. The Moving -Average method yielded AUC-values that were about 0.1 units higher for 3 kHz than for 16 kHz stimulation, because the lower number of strong responses to IFBs caused more stable reference activity and allowed the Moving-Average method to detect more DBBs. The AUC-values obtained for the detection of 3 kHz stimulus intensity decreases with the Pure-ISI method varied even more than for 16 kHz stimulation. While the method showed good performance values (AUC > 0.7) in some experiments, it failed for others.

#### 3.3.2. Extracellular Experiments

In comparison to the intracellularly recorded data, the extracellular recordings showed in general more variability between preparations. Nevertheless, for the 16 kHz experiments in principle the same response behavior was observed with both recording techniques. Consequently, also similar AUC mean values were obtained, with extracellular recordings yielding a higher variability (Figure 5A).

In contrast, the recording technique had more drastic consequences for the data obtained in response to 3 kHz stimulation. There, fewer spikes were detected in extracellularly than in intracellularly recorded data. In particular, more stimulus intensity increases (especially onsets and IBBs) failed to increase the neuronal activity (ISI-Ratio > 1) and were associated with long ISIs (ISI > 40 ms). The reason for this observed difference is the spike-sorting procedure. When recording extracellularly from AN2, also spikes from the adjacent interneuron AN1 are registered. Since this interneuron is tuned to low frequencies, 3 kHz stimulation causes a mix of spikes from both types of neurons. Hence, stricter spike sorting criteria were needed to exclude AN1 spikes, which probably also omitted some AN2 spikes.

Since the data obtained in 3 kHz extracellular recordings were in general more noisy, the robustness to noisy data can be compared based on this data set. The Moving-Average, Pure-ISI and ISI-Ratio methods showed for the 3 kHz stimulation a clear performance drop in extracellularly compared to intracellularly conducted experiments. Only the classification method was robust against the increased noise level of extracellular recordings, yielding consistently good performances for all types of experiments.

## 4. Discussion

In this study four different online methods were compared for the detection of sound intensity changes based on single spike trains recorded in the auditory interneuron AN2 of a cricket. The main result of this study is that the simple ISI-Ratio method provides stimulus change detection performance that is higher than obtained the standard Moving-Average method, and almost as high as for the complex classification method (Table 1). In contrast, the even simpler Pure-ISI method is not suitable for the detection of intensity decreases.

**Table 1:** Overview of the change point detection methods including technical aspects, performances, and applications.

		**Moving-Average**	**Pure-ISI**	**ISI-Ratio**	**Classification**
**Method**	Distribution	Symmetric	No assumption	No assumption	No assumption
	Reference data	ISIs in a preceding interval	None	Weighted previous ISI	Training data, weighted previous ISI
	Reference window	Fixed	None	Flexible	Flexible
	Number of parameters	3	2	3	3 & Training data set
	Average computation time for one parameter combination	0.0413 s	0.101 s	0.125 s	0.051 s
**Performance**	Intensity increases	+	+	+	+
	from baseline	+	++	++	++
	back to baseline	o	−	+	o
	Intensity decreases	o	−	+	+
	back to baseline	o	−−	+	++
	from baseline	+	+	o	o
**Application**	Extracellular rec.	o	o	o	+
	increases	+	+	+	++
	decreases	o	−	−	+
	Burst detection				
	Burst onset	+	++	++	++
	Burst offset	o	−−	+	++
	Pause detection				
	Pause onset	+	+	o	o
	Pause offset	o	−	+	o
	Biophysical plausibility	Not plausible	Plausible	Plausible	Not plausible

### 4.1. Comparison of Methods

It should be noted that the data set used in this study was not optimized for any of the tested methods, but represents a realistic example of available electrophysiological recordings of stimulus-triggered neuronal activity. When comparing the detection of stimulus intensity increases, all methods including the Pure-ISI method could reliably detect strong responses, usually triggered by increases from baseline stimulus intensity. One possible explanation for the success of the Pure-ISI method could be the adaptive rescaling of the auditory system (Brenner et al., 2000; Dean et al., 2005; Marsat and Pollack, 2006; Wimmer et al., 2008; Hildebrandt et al., 2011; Clemens et al., 2018), that causes activity regulation relative to mean and standard deviation of the signal statistics. Medium responses were detected best by the Moving-Average and ISI-Ratio methods. Judging by the AUC-values, the classification method outperformed all other methods, because it detected also weak responses. However, these detections were often caused by the “false” training effect, which included the stimulus autocorrelation for CP prediction. This resulted in good performances even for noisy extracellular recordings, where little information was available from the spike trains directly.

Analyzing the detection times of the putative CPs between the methods, we found that most of the changes were detected at the same spike time for all four methods. In some cases, the ISI-Ratio method detected the CPs one spike earlier than the other methods, because in strong responses the minimum ISI-Ratio occurred often one spike earlier than the minimum ISI. In some cases, the “false” training effect of the classification method resulted in a shorter detection time for weak responses.

Intensity decreases were generally more difficult to detect than intensity increases. Only the ISI-Ratio and the classification method yielded good, reliable performances for the detection of intensity decreases back to baseline in intracellular experiments. The Moving-Average method had sometimes problems to identify decreases after a strong response to an intensity increase, probably because of the high variation of the neuronal activity within the reference window. The Pure-ISI method failed for most of the intensity decreases, except when a particularly long ISI occurred, in particular because the accepted time range was often too short for this method.

In contrast to the intensity increases, the detection times of decreases differed systematically between the methods. Compared to the ISI-Ratio and classification methods, the Moving-Average identified decreases back to baseline 5–10 ms faster and the Pure-ISI method needed typically 5–20 ms longer for all stimulus changes. The classification method displayed more variable detection times then the ISI-Ratio, due to the “false” training effect. If the estimated probability for an intensity decrease was enhanced within the accepted time range, but before the neuron actually responded to the intensity change, the classification method yielded a shorter detection time than the other methods. On the other hand, if a putative CP was detected before the accepted time range, a false positive was generated and often followed by a late true positive after resetting the algorithm.

The change point detection problem is closely related to the detection of bursts and pauses in spike trains, for which several offline algorithms were published (Legéndy and Salcman, 1985; Cocatre-Zilgien and Delcomyn, 1992; Xu et al., 1999; Pauluis and Baker, 2000; Gourévitch and Eggermont, 2007; Pasquale et al., 2010; Tokdar et al., 2010; Kapucu et al., 2012; Ko et al., 2012). For estimating the applicability of our four methods for burst/pause detection, identifying responses to positive intensity steps can be construed as burst detection: burst onset (intensity increase from baseline) and burst offset (intensity decrease back to baseline). Finding negative intensity steps based on the neuronal responses can be interpreted as pause detection: pause onset (intensity decrease from baseline) and pause offset (intensity increase back to baseline; Table 1).

#### 4.1.1. Moving-Average Method

The Moving-Average method calculates the mean and standard deviation of the instantaneous spike rate in a moving interval. Consequently this method implicitly assumes that the underlying instantaneous spike rate distributions in the moving intervals are at least symmetrical and continuous. This assumption poses a potential theoretical flaw, because the instantaneous spike rate can never reach negative values.

Additionally, this method requires stable activities in reference windows of fixed size to detect changes reliably (Koepcke et al., 2016). Consequently, the optimal window size depends strongly on the spacing between the intensity changes. For the data set used in this study all positive intensity steps lasted 50 ms, resulting in short periods of stable activity and consequently low performance for the detection of DBBs. Moreover, the stimuli used in this study were separated by a fixed period. Depending on the stimulus protocols, the fixed window length assumed by this method could lead to problems for some data sets, in particular since stimulus changes occur at unpredictable times in a natural environment.

The Moving-Average method is not used as a typical burst/pause detection method, because it requires a continuous time series rather than discrete spike times. In this study, the Moving-Average was shown to be suitable for detecting the onsets of bursts and pauses, but not the offsets.

#### 4.1.2. Pure-ISI Method

The Pure-ISI method (section 2.3.2, Figures 2A1–A3) compares the adjusting ISI with a given threshold. It is comparable to the method of Ratnam et al. (2003), where a given threshold is applied continuously to a PSTH to detect weak sensory signals.

Response to intensity increases could be detected earlier than for intensity decreases and the response latencies had narrower distributions. This finding agrees with faster onset than offset responses found in many systems (Di Lollo et al., 2000; Phillips et al., 2002; Humphreys et al., 2006; Scholl et al., 2010; Ramamurthy and Recanzone, 2017).

AN2 responses to strong intensity increases typically trigger ISIs in a range of 2.5–4 ms. According to (Marsat and Pollack, 2006), ISIs shorter than 6.5 ms correspond to bursting behavior, which they showed to be suitable for feature detection of ultrasound stimulation of the AN2 neuron in *Teleogryllus oceanicus*.

The Pure-ISI method is similar to published burst onset detecting algorithms (Legéndy and Salcman, 1985; Cocatre-Zilgien and Delcomyn, 1992; Kepecs and Lisman, 2004; Marsat and Pollack, 2006) in comparing the ISI with a fixed threshold. However, the studies differed considerably in their methods analyzing the ISI distribution for obtaining a suitable threshold (Legéndy and Salcman, 1985; Ramakers et al., 1990; Cocatre-Zilgien and Delcomyn, 1992; Marsat and Pollack, 2006; Gourévitch and Eggermont, 2007; Pasquale et al., 2010; Ko et al., 2012). As expected, the Pure-ISI method very successfully detected burst onsets and also was suitable to detect pause onsets.

#### 4.1.3. ISI-Ratio Method

The ISI-Ratio method introduced in this study assumes that the intensity changes are reflected in the ratio of the actual and the previous activity. Several possible modifications of the ISI-Ratio method are described in the Supplementary Material. Analyzing a (likelihood) ratio to detect and estimate CPs is an established approach (Gombay, 2000; Kawahara and Sugiyama, 2012; Zhou et al., 2014), which is applied e.g., in the CUSUM method. The advantage of this approach is that it is easier to estimate the ratio than both distributions (Kawahara and Sugiyama, 2012).

In our proposed method, the weighted previous ISI is used for calculating the ratio. Extending the reference ISI to the second last ISI ensured a better estimation of preceding neuronal activity for medium or high neuronal activities. However, if the activity was low, the second last ISI could not reflect the directly preceding activity of the neuron. The weight only shows a noticeable effect for detecting intensity decreases (Figure 3). The main reason is that AN2 neurons adapt to sound intensities (Wimmer et al., 2008), resulting in longer ISIs. Directly after an intensity decrease the second last ISI could be shorter than the previous ISI. Therefore, the ratio can be higher when the second last ISI is included, resulting in improved detection rate and time. In contrast, the weight has no strong impact on the performance of intensity increases, because of the bursting behavior of the neuron. Bursts cause short ISIs and small ISI-Ratios. Hence, the exact estimation of the preceding activity is far more important for intensity decreases than increases.

The ISI-Ratio method is similar to burst detection methods, even though published methods are rather based on the distribution of absolute ISI values than the ISI-Ratio (Cocatre-Zilgien and Delcomyn, 1992; Kepecs and Lisman, 2004; Pasquale et al., 2010; Ko et al., 2012). In our study, the ISI-Ratio method was good at detecting burst onsets and offsets, as well as pause offsets (Table 1). A combination of ISI-Ratio and actual ISI could be a good approach for detecting bursts and pauses in a spike train.

#### 4.1.4. Classification Method

The classification method learns to interpret a simplified combination of the adjusting ISI and the weighted previous ISI. It requires the same reference ISI as the ISI-Ratio method, leading to the same considerations as discussed before. Additionally, a training data set is required to learn the neuronal responses to different stimulus conditions. A clear advantage of this learning is that this method is more robust toward misclassified spikes in extracellular recording. However, falsely detected or missing spikes could lead to uncommon class combinations, which are not recognized as possible stimulus changes.

For our data set, good performances were achieved when applying between 8 and 15 categories. If more than 15 categories were used, the performance dropped in many experiments due to overfitting. We restricted the number of categories to 10 to account for our relatively small amount of data.

Since ISIs directly after increases from baseline and onsets are often very short, the borders of the categories were chosen to be logarithmically spaced (Figure 3). Linearly spaced class borders would assign all short ISIs (e.g., < 6.5 ms) to the same category. Logarithmically spaced borders require prior knowledge about minimum and maximum ISI. Alternatively, the class borders could be chosen based on the quantiles of the ISI distribution in the training data set. Applying this modification enhanced the detection of low responses to in- and decreases.

As it is typical for unsupervised learning algorithms, the classification method sometimes falsely identified a spike pattern as the neuronal response to a stimulus change. “False” training happened if a certain neuronal activity pattern repeatedly appeared before a response to a stimulus change started. Since by definition stimulus intensity decreases back to baseline following a positive intensity step 350 ms earlier, the classification algorithm learned to identify the response to this increase, rather than the reduced activity following the stimulus decrease. This would not happen for a more irregular stimulus protocol. The false learning could be reduced by shifting the training interval for the decreased intensity to 20–65 ms instead of 10–55 ms after each change, but assuming a longer response latency on the other hand can lead to missing the onset of strong responses to stimulus changes. Another possible approach that might reduce the problem of false training would be to use relative rather than absolute ISI changes for training.

In principle, the classification method could be used for the purpose of burst and gap detection. Here, the classification method was found to be very well-suited for detecting burst onsets and offsets. Medium performances were achieved for the detection of pause onsets and offsets.

### 4.2. Biological Plausibility

“All models are wrong, but some are useful.”

-(George E. P. Box, 1979)

While in this study the main focus was on the analysis of neuronal responses from the perspective of a data analyzer, the complementary question is how a nervous system could detect CPs. Unfortunately, it is difficult to address this question experimentally. That would require the synchronous recording of several neuronal responses and their postsynaptic effects, ideally complemented by measurements of the behavioral reactions to stimulus changes. Here, we will shortly comment on the question, if the four CP detection methods of this study could in principle be implemented by biological nervous systems.

It is unrealistic that a simple nervous system as the cricket auditory system could perform the tasks which are essential for the Moving-Average method and the classification method. Otherwise the nervous system would have to calculate and remember the variation of the previous neuronal activities. In contrast, change detection based on single ISIs like in the Pure-ISI and ISI-Ratio method is conceivable. It is known that many synapses are unreliable in transferring single presynaptic spikes to the postsynaptic neuron (Lisman, 1997). Fast successive spikes, especially bursts, ensure that the information transfer is more reliable. For the Pure-ISI method, detection of stimulus onsets or increases resulting in short ISIs could be implemented by a postsynaptic neuron that needs two overlapping EPSPs to reach the spike threshold. The threshold of the Pure-ISI method would refer to the maximum time between two EPSPs that would still trigger a postsynaptic spike. If the refractory period in the postsynaptic neurons is longer than in the presynaptic neuron, subsequent presynaptic spikes could be suppressed, leading to a single spike indicating a stimulus increase.

Compared to the Pure-ISI method, the ISI-Ratio method requires a more flexible maximum time between two EPSPs that elicit a postsynaptic spike. The interaction between the fast postsynaptic integration time constant and a slower process, e.g., of sodium channel deactivation may result in a relative threshold for the ISI. Hence, the more time past since the previous postsynaptic spikes, the easier it is for a pair of EPSPs to elicit a postsynaptic spike, allowing longer ISIs as cues for change detection.

The detection of stimulus offsets or decreases based on the principles of the Pure-ISI and ISI-Ratio method would require postsynaptic processing in a separate pathway. Intensity decreases could be detected by a spontaneously active neuron that is inhibited by the presynaptic neuron. Frequent presynaptic spiking could suppress spontaneous postsynaptic activity. A reduced presynatic spiking activity could release postsynaptic spiking. The principles of the ISI-Ratio method can be conveyed to the effect of rebound spikes (Schulz and Reynolds, 2013). The stronger the postsynaptic membrane is hyperpolarized, the higher is the probability of rebound spikes after the hyperpolarization ends. Hence, assuming different pathways for the detection of stimulus in- and decreases, the core idea of the Pure-ISI and ISI-Ratio method could be implemented already by a minimal system of one presynaptic and two postsynaptic neurons.

### 4.3. When to Use Which Method?

Although, it can widely be found in the literature, the Moving-Average method is the method which is least probable to be implemented by biological nervous systems, since it requires long periods of stable neuronal activity. Hence, it is vulnerable to spike sorting errors and can only be applied successfully to responses to stimulus changes, which are separated by a rather long time, as could be seen in this study from the low performance of detecting activity decreases after rapid changes.

If only bursts or similarly strong responses to stimulus changes need to be detected, the Pure-ISI method can be interpreted in terms of the biophysics of spike generation and is easiest to apply. Alternatively, one of the other published burst detection methods could be used for data analysis (e.g., Legéndy and Salcman, 1985; Ramakers et al., 1990; Cocatre-Zilgien and Delcomyn, 1992; Gourévitch and Eggermont, 2007; Pasquale et al., 2010; Ko et al., 2012). However, the Pure-ISI method is not a proper choice for identifying small activity increases and/or activity decreases.

The ISI-Ratio method performs for recordings with a high signal-to-noise ratio and a medium to high mean spike rate at least as well as the other methods, including the much more complex classification method. This method provides a biologically realistic, yet simple strategy for CP detection based on relative activity changes rather than absolute values. The principle of the ISI-Ratio method could not only be applied successfully by data analysts, but might resemble the biophysical processes underlying CP detection in the actual nervous system of the cricket.

The classification method is a powerful tool to employ statistical differences between different stimulus situations, yielding high performances for the detection of most stimulus changes. Classification can be the data analysis method of choice, in particular for extracellular recordings with potential spike sorting errors. However, it also is the method requiring training data in addition to three parameters, and being most vulnerable to overfitting and false training effects, leading to false positive detections. From a biological perspective, the classification method can be compared to evolutionary processes, optimizing neuronal response to common stimulus contexts, but does not refer to biophysical processes occurring in a single neuron.

Finding the optimal data analysis method for a specific research question depends strongly on the stimulation time course and the neuronal response type under study. Simple nervous systems like the cricket's might employ quite simple biophysical mechanisms for fundamentally important tasks like stimulus change detection. Hence, applying biologically more realistic data analysis approaches, like the ISI-Ratio method introduced in this study, might be an alternative to the commonly used data analysis techniques.

## Data Availability Statement

Publicly available datasets were analyzed in this study. This data can be found here: a https://web.gin.g-node.org/LenaKoepcke/Spike_trains_stimulus_change_point_detection.

## Author Contributions

LK developed and implemented the methods, analyzed the results, and wrote the paper. KH performed the experiments, developed the methods, and improved the paper. JK designed the study, developed the methods, and wrote the paper.

### Conflict of Interest

The authors declare that the research was conducted in the absence of any commercial or financial relationships that could be construed as a potential conflict of interest.

## Abbreviations

AN: Ascending neuron
ISI: Interspike interval
ISR: Instantaneous spike rate
PSTH: Peri stimulus time histogram
CP: Change point
TP-Rate: True positive rate
FP-Rate: False positive rate
ROC: Receiver operating characteristic
AUC: Area under the curve
IFB: Intensity increase from baseline
IBB: Intensity increase back to baseline
DFB: Intensity decrease from baseline
DBB: Intensity decrease back to baseline.
