# Supplementary material for: Online Detection of Multiple Stimulus Changes Based on Single Neuron Interspike Intervals
Source: Front Comput Neurosci. 2019 Oct 1;13:69. doi: 10.3389/fncom.2019.00069 (PMC6779812; doi:10.3389/fncom.2019.00069)
Supplement: Supplementary file 1 [file Data_Sheet_1.pdf]

frontiers

Supplementary Material: Online detection of multiple stimulus changes based on single neuron interspike intervals

1 ACCEPTED TIME RANGE AND TESTED THRESHOLDS

The accepted time range was determined by estimating the distribution of the response latencies. For the same animals, which were analyzed with the CP detection method, the distribution of the response latencies was estimated based on responses to an additional protocol.

1.1 Stimulus protocol for estimating response latency

Only one constant sound intensity was presented for 500 ms. The recording of neuronal activity started 750 ms before and ended 750 ms after the stimulation. Depending on the absolute threshold of hearing, the sound intensity varied for each animal between 45 and 98 SPL. Each animal was stimulated with 5 to 40 different sound intensities which were repeated between 6 and 15 times. In total 439 experiments could be analyzed, of which 134 were recorded intracellularly (16 kHz: 60, 3 kHz: 74, in total 9 animals) and 305 extracellularly (16 kHz: 245, 3 kHz: 60, in total 12 animals).

1.2 Estimating the distribution of the response latency

The latencies for intensity in- and decreases were estimated based on a peristimulus time histogram (PSTH) averaged over all trails. The response latencies were estimated with the half-height method (Levakova et al., 2015). If the method could not detect any change in the neuronal activity or the activity did not differ compared to the spontaneous activity, the experiment was discarded. The latencies of onsets and offsets were analyzed independently. In Table S1 three quantiles (2.5%, 97.5%-quantile and the median) of the empirical latency distribution are listed. The distributions are divided into stimulus onsets/offsets and further divided into 16 kHz/3 kHz stimulation.

1.3 Definition and discussion of the accepted time range

The 2.5%- and the 97.5%-quantile ($Q_{0.025}, Q_{0.975}$) of the estimated distributions were used as guide to determine the accepted time range after a stimulus change T_{true}, where a CP was classified as true positive (TP). We set the beginning of T_{true} to 2.5%-quantile and the end to the 97.5%-quantile plus 5 ms. The 5 ms were added because: (1) the methods might require some time to detect changes and (2) the response latencies to intensity in-/decreases might appear later than for stimulus onset/offset.

One disadvantage of this approach is that the stimulus protocol contained different types of stimulus changes, which induced different response latencies. To weaken this effect the maximal allowed detection time was extended to 5 ms after the 97.5% quantile of the estimated distribution. This extension might be too short for some stimulus changes especially for DFBs. The ISI-Ratio method could reliable detect these changes about 75-80 ms after the intensity decrease. Therefore, a shift of more than 20 ms would be suitable.

The accepted time range for true positives after intensity increases was set to [10, 40] ms and after intensity decreases to [15,55] ms

	$Q_{0.025}$	$Q_{0.5}$	$Q_{0.975}$	Number of estimated latencies
Onset	10 ms	16 ms	35 ms	439
16 kHz	11 ms	16 ms	29 ms	305
3 kHz	9 ms	19 ms	37 ms	134
Offset	15 ms	22 ms	50 ms	356
16 kHz	14 ms	23 ms	50 ms	259
3 kHz	15 ms	24 ms	51 ms	97

Table S1. Estimated distribution of the response latency (in milliseconds) according to sound intensity onsets and offsets. The latencies were estimated in different animals. The underlying data contained intra- and extracellularly recordings. The number of the analyzed experiments is shown in the last column. The 2.5% and the 97.5%-quantiles are used for the definition of true and false positive change points.

	**Intensity Increases**		**Intensity Decreases**	
	Parameter Space of θ_{in}	Tested values	Parameter Space of θ_{de}	Tested values
Moving-Average	$]0, \infty[$	300 values between 0 and 20, with shorter distances between 0 and 3	$]0, \infty[$	the same values as for the increases
Pure-ISI	$]0, \infty[$	600 values between the minimum and maximum ISI, with shorter distances around the minimum	$]0, \infty[$	600 values between minimum and maximum ISI with smaller distances around the median ISI
ISI-Ratio	$]0, 1[$	500 values between 0 and 1 with smaller distances around 1	$]1, \infty[$	500 values greater than 1 and smaller than 100, with shorter distances between 1 and 15
Classification	$[0, 1]$	200 values between 0 and 1	$[0, 1]$	200 values between 0 and 1

Table S2. Parameter space and the tested values for the threshold parameters θ_{in} and θ_{de} for every method. The tested values were used to generate the ROC-curve and to calculate the AUC-values

1.4 Tested threshold values

For every applied method the tested parameters are listed in Table S2. The results of the tested parameters are the basis of constructing the ROC-curve and the resulting AUC-value.

2 DISCUSSION ABOUT TECHNICAL ASPECTS OF EVALUATION CRITERIA

2.1 Evaluation criteria

The evaluation performance of the methods relies strongly on the definition of the accepted time range and the definition of the FP-Rate and the ROC curves. The ROC curve is a graphical tool and its corresponding AUC-value a statistical technique to analyze the effect of a threshold criterion on the false and true positive rates (FP-Rate, TP-Rate). This technique is commonly used in several research fields, including psychophysics (detection of weak signals, (Ratcliff et al., 1992)), medicine (evaluation of diagnostic tests, (Zweig and Campbell, 1993)), and machine learning (evaluation of classification algorithms, (Spackman, 1989)). Several alternative approaches to determine an optimal threshold based on the shape of the ROC-curves can be found in the literature (Habibzadeh et al., 2016; Akobeng, 2006). One way is to optimize the Youden's index (TP-Rate + TN-Rate -1) (Youden, 1950) or to minimize the distance to the upper left corner (point (0,1)) (Delong et al., 1988). However, ROC and AUC were originally developed to evaluate a

binary classifier by varying its discrimination threshold. The analysis of time series, in which data points are not independent from each other, requires modification of these methods.

FP-Rate and ROC curve

Two challenges for calculating ROC for our data sets were that the neuronal activity depended strongly on the previous activity, and that the false positive rate was not clearly defined. The stimulus time course determined the time ranges, in which putative CPs identified by the four methods were classified as true positives. However, within these ranges true negatives and false positives could also appear. Hence, we defined a normalized FP-rate : FP-Rate $= \frac{\#\text{FP}}{D/|T_{\text{accept}}|-n_{\text{in}}}$ (see Eq. 21). Hence, in contrast to the theory of ROC-curve, FP-rates greater than 1 could occur in our application. For the ISI-Ratio method, all FP-rates were smaller than 1 when the activity did not in- or decrease during a period longer than 40 ms. To solve these problems, we ignored pairs of FP-rate and TP-rate when the FP-rate was higher than 1. If the highest FP-rate was smaller than 1, an additional pair was created. The FP-rate of this pair was set to 1 and the TP-rate to the highest achieved value. It was possible that the TP-rate did not increase continuously because this rate was averaged over different trials. To avoid this effect, we fitted the values with an exponential function $\frac{a(1-b)}{exp(xc+d)}$. No significant difference could be found between the fitted and the original AUC-values.

Alternative evaluation criteria

An alternative evaluation criterion could be for example to maximize a weighted function. Compared to the ROC-values, the advantages of e.g. the approach TP-Rate $\cdot$ factor $-$ FP-Rate are that no modification (reset) of the algorithm is necessary, and that the FP-Rate does not have to be normalized, because the FP-Rate is not restricted to a certain range. The disadvantages of this alternative criterion are that the proportion between TP- and FP-Rate have to be set in the beginning and that only one point of the ROC curve is analyzed.

2.2 Modifications of the ISI-Ratio method

Several modifications could be applied to the ISI-Ratio method to allow optimal change detection in specific data sets. E.g., neuronal activity can be estimated alternatively by using all ISIs in an interval of fixed length, like the window in the Moving-Average method. The reference ISI could then be calculated by a weighted mean. Another possible modification is to assume an underlying Poisson process. Under this assumption the ISIs are exponentially distributed. The weighted previous ISI could be used to estimate the exponential distribution's rate parameter. This distribution is compared to the adjusting ISI to calculate the probability of a change point. Alternatively to using the ratio assumption, it could be assumed that intensity changes are reflected best by the linear difference between adjusting and weighted previous ISI. When we tested this additive assumption, the modified method performed very similar to the original ISI-ratio method, but decreases back to baseline and offsets were detected on average 10 ms later.

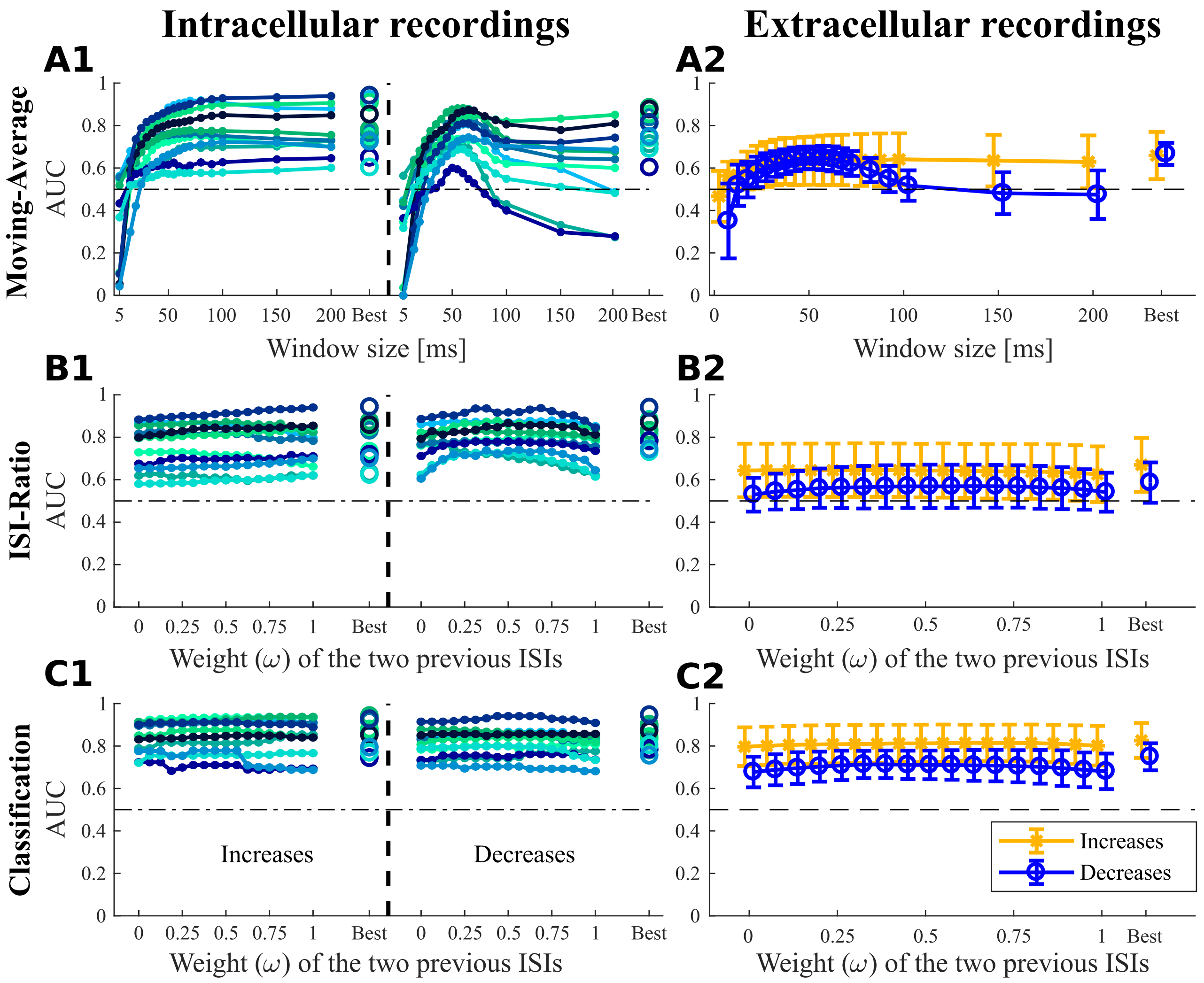

Figure S1. For 3 kHz stimulation, influence of the additional parameters on the AUC-values for the Moving-Average **A1-2**, ISI-Ratio **B1-2** and Classification method **C1-2**. **A1, B1, C1** For the 9 intracellular recordings of responses to 16 kHz stimulation, the AUC-values are illustrated separately. **A2, B2, C2** The performances obtained for the 43 extracellular recordings are summarized by means (circles) and standard deviations (error bars) over experiments. For the Moving-Average method **A1-2**, the influence of the moving average window size on the AUC-value is shown. For the ISI-Ratio **B1-2** and **C1-2** Classification, the weight of the the two previous ISIs was varied to analyze their influence on the AUC-value. 17 weights between 0 and 1 were tested for calculating the previous ISI. A weight of 0 indicates no influence of the second last ISI, whereas the last ISI had no influence if the weight was equal to 1.

REFERENCES

Akobeng, A. K. (2006). Understanding diagnostic tests 3: receiver operating characteristic curves. *Acta Paediatr.* 96, 644–647. doi:10.1111/j.1651-2227.2006.00178.x

Delong, E., Felong, D., and Clarkepearson, D. (1988). Comparing the areas under 2 or more correlated receiver operating characteristic curves - A nonparametric approach. *Biometrics* 44, 837–845. doi:10.2307/2531595

Habibzadeh, F., Habibzadeh, P., and Yadollahie, M. (2016). On determining the most appropriate test cut-off value: the case of tests with continuous results. *Biochem. Medica* 26, 297–307. doi:10.11613/

BM.2016.034

Levakova, M., Tamborrino, M., Ditlevsen, S., and Lansky, P. (2015). A review of the methods for neuronal response latency estimation. *BioSystems* 136, 23 – 34. doi:http://dx.doi.org/10.1016/j.biosystems.2015.04.008. Selected papers presented at the Eleventh International Workshop on Neural Coding, Versailles, France, 2014

Ratcliff, R., Gronlund, S., and Sheu, C. (1992). Testing global membory models using ROC curves. *Psychological Review* 99, 518–535. doi:{10.1037/0033-295X.99.3.518}

Spackman, K. A. (1989). Signal detection theory: Valuable tools for evaluating inductive learning. In *Proceedings of the Sixth International Workshop on Machine Learning*, ed. A. M. Segre (San Francisco (CA): Morgan Kaufmann). 160 – 163. doi:https://doi.org/10.1016/B978-1-55860-036-2.50047-3

Youden, W. J. (1950). Index for rating diagnostic tests. *Cancer* 3, 32–35. doi:10.1002/1097-0142(1950)3:1⟨32::AID-CNCR2820030106⟩3.0.CO;2-3

Zweig, M. and Campbell, G. (1993). Receiver-Operating Characteristic (ROC) Plots - A fundatmental evaluation tool in clinical medicine. *Clinical Chemistry* 39, 561–577
